# The Effect of Tart Cherry on Sleep Quality and Sleep Disorders: A Systematic Review

**DOI:** 10.1002/fsn3.70923

**Published:** 2025-09-16

**Authors:** Fateme Barforoush, Sara Ebrahimi, Maryam Karimian Abdar, Shabnam Khademi, Nava Morshedzadeh

**Affiliations:** ^1^ Student Research Committee Kerman University of Medical Sciences Kerman Iran; ^2^ Institute for Physical Activity and Nutrition, School of Exercise and Nutrition Sciences Deakin University Geelong Australia; ^3^ Department of Nutrition, Faculty of Public Health Kerman University of Medical Science Kerman Iran

**Keywords:** inflammation, neurotransmitter, review, sleep disorders, sleep quality, tart cherry

## Abstract

This systematic review examined the association between tart cherry consumption and sleep disorders. Tart cherries contain active compounds such as melatonin and anthocyanins that may be effective in improving sleep quality. The aim of this study was to investigate the effect of tart cherry consumption on sleep quality and duration, sleep efficiency, melatonin levels, inflammation, and oxidative stress. A systematic review was conducted in PubMed, Google Scholar, ScienceDirect, Scopus, and EMBASE databases in January 2025 without time restrictions. English‐language interventional studies that examined the effect of tart cherry consumption on sleep, inflammation, and neurotransmitter synthesis were included, and studies related to other chronic diseases were excluded. In total, seven interventional studies were included in the review. Three studies reported significant improvements in sleep indicators such as sleep duration, sleep efficiency, or sleep onset time. Three studies also reported an increase in melatonin levels after tart cherry consumption. Two studies also reported a decrease in inflammatory markers such as CRP and MDA. However, there were large differences in dose, duration of intervention, and characteristics of the participating populations. Although tart cherry consumption may be effective in improving sleep quality, reducing inflammation, and increasing antioxidant capacity, the available evidence is still limited and heterogeneous. To demonstrate clinical efficacy and clarify the mechanisms of action, high‐quality, carefully designed clinical trial studies in diverse populations are essential.

## Graphical abstract

Influence of tart cherry on biomarkers of inflammation and oxidative stress. This image points to the aims of this study possible mechanisms by which tart cherries (including both sweet and sour varieties) can improve sleep cycles. Compounds such as melatonin, serotonin, tryptophan, and phytochemicals in tart cherries with antioxidant and anti‐inflammatory effects (via the JAK/STAT pathway) may improve sleep quality by reducing inflammation and oxidative stress. These compounds are effective in better regulating sleep by enhancing different stages of sleep, especially non‐REM and REM stages.

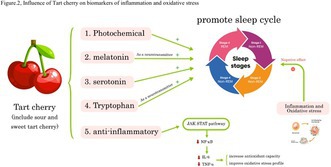

**Abbreviations**
ASBQ: athlete sleep behavior questionnaire
ASS: athlete sleep screening questionnaire
CBT: cognitive behavioral therapy
CNS: central nervous system
DFA: difficulty falling asleep
DMS: difficulty maintaining sleep
EMA: early morning awakening
LE: life expectancy
NTR: nighttime recharge
OSA: obstructive sleep apnea
PSQI: Pittsburgh Sleep Quality Index
REM: rapid eye movement
RLS: restless legs syndrome
ROS: reactive oxygen species
SWS: slow‐wave sleep
TLR4: toll‐like receptor 4
TNF: tumor necrosis factor
WHO: World Health Organization

## 1 Introduction

Sleep is a vital physiological process that is essential for maintaining physical and mental health. Sleep accounts for approximately one‐third of the human lifespan and plays a key role in cognitive function, mood regulation, immune system enhancement, and memory consolidation (Helvig et al. 2016; Huyett et al. 2021; Reis et al. 2018).

While the sleep cycle includes several stages from light sleep to rapid eye movement (REM) sleep (Memar and Faradji 2017), sleep disruption can lead to serious health consequences, including cardiovascular disorders, obesity, hypertension, and type 2 diabetes (Irwin 2019; Reis et al. 2018; Zhao et al. 2020).

Sleep disorders such as insomnia and obstructive sleep apnea (OSA) affect a significant portion of the population and negatively impact quality of life (Binks et al. 2020). Evidence suggests that nutrition may play an important role in sleep regulation through a variety of mechanisms (Binks et al. 2020; Peuhkuri et al. 2012). On the other hand, it seems that nutrients can have a potential impact on sleep quality through anti‐inflammatory mechanisms (Abdollahi et al. 2021; Niseteo et al. 2024); nonetheless, some compounds that appear to have anti‐inflammatory effects have been shown to have anti‐inflammatory effects in some studies (Morvaridi et al. 2020). Specific dietary components, including anti‐inflammatory agents, melatonin‐enhancing foods, and dietary sources of tryptophan and serotonin, can influence sleep quality (Han et al. 2022).

Many research studies have focused on different impacts of nutrients when it comes to the prevention and treatment of sleep disorders. Growing scientific evidence suggests that consuming fruits and vegetables such as tart cherries can play an effective role in reducing the risk of chronic diseases due to their anti‐inflammatory and antioxidant compounds (Martin et al. 2018).

In recent years, tart cherry juice has attracted the attention of many researchers due to its anti‐inflammatory properties (Chai et al. 2019). This fruit can play an effective role in improving inflammatory conditions by reducing inflammatory processes and inhibiting oxidative stress (Bell et al. 2014). Anthocyanins, which are the predominant bioactive compounds in tart cherries, are able to inhibit the enzyme cyclooxygenase‐2 (COX‐2), which is involved in inflammation (Keane et al. 2018); a mechanism that is likely one of the main reasons for the anti‐inflammatory effects of tart cherry juice.

In the meantime, tart cherries ( *Prunus cerasus* ) have been increasingly recognized for their compounds, such as melatonin. These compounds may play an effective role in improving sleep quality by increasing circulating melatonin levels and reducing inflammatory processes and oxidative stress. Melatonin, known as the master regulator of the body's circadian rhythms, is naturally present in tart cherries and may help facilitate sleep onset and increase sleep duration (Hillman et al. 2022). Also, the anthocyanins in this fruit can be effective in improving sleep disorders associated with chronic inflammation due to their strong antioxidant and anti‐inflammatory properties (Nohara et al. 2018).

Despite the beneficial properties of tart cherries, this fruit has been proposed as a promising nutritional intervention for improving sleep quality. However, despite several preliminary studies investigating this association, a more detailed understanding of the effects and underlying mechanisms requires the collection and analysis of coherent scientific evidence.

Accordingly, the present systematic review was designed to examine the existing evidence on the effects of tart cherry consumption on various aspects of sleep, including sleep onset time, nighttime sleep duration, and related biomarkers such as inflammatory markers. The review also examined diverse populations to better assess the generalizability of the results to different groups.

It is hypothesized that consuming tart cherries can enhance serum melatonin levels and antioxidant capacity in humans, promoting better sleep. This review aims to explore the effects of tart cherry and its compounds on sleep quality while also clarifying the potential mechanisms involved in the effect of tart cherry on various aspects of sleep.

## 2 Materials and Method

### 2.1 Search Strategy

**FIGURE 1:**
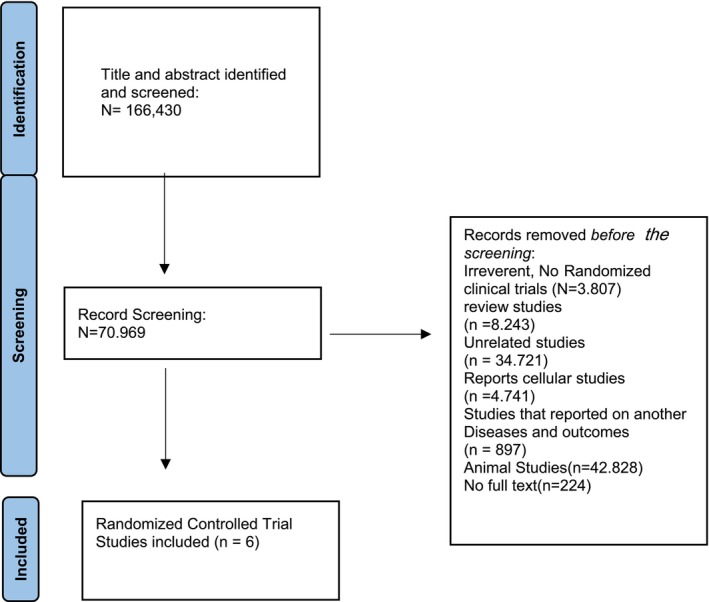
PRISMA flowchart of a systematic review of tart cherry on sleep quality and sleep disorders.

This systematic review was conducted according to the Preferred Reporting Items for Systematic Reviews and Meta‐Analyses criteria. The criteria were confirmed using the PRISMA checklist for completeness of systematic review findings (Figure 1) (Page et al. 2021). The databases, including PubMed, MEDLINE, Google Scholar, ScienceDirect, Scopus, and EMBASE, published in peer‐reviewed journals in English with no time restriction, were searched up to January 2025.

The Boolean operators “AND” and “OR” for published peer‐reviewed studies were undertaken by two researchers within the electronic databases. The following MeSH and non‐MeSH terms were used in our search strategy: “Sour Cherry” OR “Tart Cherry” OR “dwarf cherry” OR “Prunuscerasus” OR “morello cherry” OR “amarelle cherry” OR “Montmorency cherry” OR “cherry[tiab] OR cherries[tiab] OR Montmorency[tiab] OR”, *Prunus cerasus* “[tiab] OR cherries[tiab] OR”, *Prunus avium* “[Mesh] AND sleep quality[tiab] OR sleeping disorders[tiab] OR Sleep [tiab] OR sleep onset latency [tiab] OR total sleep time[tiab] OR Insomnia[tiab] OR obstructive sleep apnea[tiab]”.

### 2.2 Data Extraction

Two researchers independently extracted the information, including the main topics, aim, study design, methodology, interventions, variables, and other primary outcomes using a data extraction template.

### 2.3 Inclusion and Exclusion Criteria

Randomized controlled trials (RCTs) and quasi‐randomized controlled trials (qRCTs), uncontrolled studies and controlled trials without randomization methods, studies published in peer‐reviewed journals in English that investigated the effect of tart cherry intake (any type of intervention such as power, juice beverage, and combination supplements containing tart cherries) on main outcomes sleep quality, sleeping disorders, sleep, sleep onset latency, total sleep time, insomnia, and OSA were included. The participants of this study were healthy individuals without age or gender restrictions.

Participants with diseases (such as metabolic syndrome, inflammatory disease, cancer, and carcinoma), non‐English language studies, and duplicate studies were excluded. The data extracted from each study include the first author, country of origin, study duration, sample size, biological specimen collected, and biomarkers measured. Any discrepancies in the interpretation and data extraction were resolved by discussion.

### 2.4 Methodological Quality Assessment

The quality of the included RCTs was evaluated using the Cochrane Risk of Bias 2 tool (Higgins et al. 2019). This tool examines the seven domains, including random sequence generation, allocation concealment, blinding of participants and personnel, blinding of outcome assessment, incomplete outcome data, selective outcome reporting, and other sources of bias. According to the Cochrane handbook, each domain is rated as having a “low,” “high,” or “unclear” risk of bias. Based on these ratings, the overall quality of each study was classified as poor, fair, or good. Specifically, a study is categorized as having a high risk of bias if two domains are rated as high or three domains are rated as unclear. If one domain is rated as high or two domains are unclear, the study is considered to have a fair risk of bias. Any other combination is classified as a low risk of bias. Two independent investigators assessed the quality of the selected studies. In cases of disagreement, a third reviewer provided the final judgment, and no difference was seen in the evaluation results with the third person's judgment.

### 2.5 Diet and Tart Cherry

Nutritional interventions, including high‐carbohydrate, high‐glycemic‐index meals, melatonin, tryptophan‐rich protein, sour cherry juice, kiwi, and micronutrients, have been shown to influence sleep quality (Peuhkuri et al. 2012). However, the nutritional mechanisms underlying sleep regulation are complex (Hirshkowitz et al. 2015). Various metabolites play a key role in sleep regulation by influencing other sleep‐related mediators (Han et al. 2022). A diet rich in fruits, vegetables, and whole grains has shown positive effects on sleep (Sejbuk et al. 2022). One of the compounds that directly affects sleep is caffeine, which reduces sleep duration and quality while increasing the time of sleep induction (Tahara and Shibata 2014).

Flavonoids are bioactive, nutritional, and functional compounds with polyphenolic structures found in various sources, including fruits, vegetables, tea, dark chocolate, and certain beverages (Mutha et al. 2021). Flavonoids are classified into six subclasses based on variations in the heterocycle (ring C): isoflavones, flavanols, flavan‐3‐ols, flavanones, flavones, and anthocyanins (Pietta et al. 2003). Extensive studies have demonstrated the beneficial effects of flavonoids, including antimicrobial, antioxidant, and anti‐inflammatory effects (Fidelis et al. 2014; Peluso et al. 2015).

Tart cherries are low‐calorie fruits that are rich in essential nutrients, including vitamins A, C, and polyphenols (Hussain et al. 2021). Cherries are typically classified into two types: sweet types ( *Prunus avium* ) and sour types ( *Prunus cerasus* ). It has been shown that sour cherries contain higher levels of anthocyanins and phytochemicals, which contribute to their antioxidant and anti‐inflammatory effects (Aglar et al. 2019).

Tart cherries are a great source of phytochemicals and have been shown to have significant health‐promoting properties (Wang et al. 2021). They contain substantial amounts of melatonin, serotonin, and tryptophan, which accentuates their potential sleep‐promoting properties (Delgado et al. 2012). Tart cherries include a high level of melatonin, which may help regulate the human sleep–wake cycle and internal temperature to induce sleep. Furthermore, tart Montmorency cherries ( *Prunus cerasus* ) are abundant in various phytochemicals that provide numerous health advantages, including improved sleep quality in individuals with late‐life insomnia (McCune et al. 2010; Rosado et al. 2017).

Jack N. Losso et al. reported that consumption of cherry juice (240 mL 2 times/day) for 2 weeks can lead to an increase in sleep time and sleep efficiency in adults over 50 years old (Losso et al. 2018).

Tart cherry intake may improve the sleep–wake cycle. A study showed that 30 mL of tart cherry juice supplementation for 7 days increased bedtime, total sleep time, sleep efficiency, and disturbed sleep in healthy individuals (Howatson et al. 2012).

A study on 19 female hockey players investigated the effect of tart cherry juice on sleep quality. The PSQI was used to measure sleep quality. An athlete sleep behavior questionnaire (ASBQ) and the athlete sleep screening questionnaire (ASSQ) were used to measure the sleep quality level. The main findings were that consumption of 200 mL of tart cherry juice in the morning and another 200 mL in the evening on the first 2 days, followed by a final 200 mL intake on the 3rd day, increased total bedtime, wake after sleep onset, and movement index variables (Chung et al. 2022). However, current studies report conflicting results, which may be attributed to variations in the intervention period or the assessment of sleep parameters. A randomized, placebo‐controlled study found that taking 1000 mg of cherry in Night Time Recharge (NTR) for 7 days had no significant effect on sleep parameters, while it had positive effects on sleep latency. These findings are in contrast to other studies, which found that cherry supplementation has a significant effect on sleep parameters (Simper et al. 2019).

A pilot study aimed to determine whether a tart cherry juice blend is associated with sleep disorders. This study included 16 participants who experienced a typical bedtime between 9:00 P.M. and 12:00 A.M. The sleep problem, occurring more than three nights per week for at least 6 months, was considered a sleep disorder. The results suggest that the tart cherry juice blend has beneficial effects on sleep disorders (Pigeon et al. 2010).

A randomized, repeated‐measures crossover with a double‐blind deception design included nine males and seven females and demonstrated that tart cherries reduced sleep onset latency, increased total sleep duration, and sleep efficiency, leading to reduced perceptions of morning sleepiness (Pigeon et al. 2010).

The evidence shows that other food compounds can have similar effects on total sleep time, sleep onset, duration, and sleep quality. A study on 95 men examined the impact of fatty fish on sleep patterns and showed that Atlantic salmon three times a week for 5 months increased sleep latency (Hansen et al. 2014). Another study demonstrated that 100 g of *Lactobacillus helveticus* fermented milk causes sleep improvement in healthy elderly individuals (Yamamura et al. 2009). Hsiao‐Han Lin found that consuming two kiwifruits 1 h before bedtime each night for 4 weeks improved sleep onset, total sleep time, and sleep efficiency in individuals aged 20–55 years (Lin et al. 2011); on the other hand, Noorwali E et al. showed that higher kiwi consumption was associated with reduced sleep duration in English women, although this reduction was considered small and clinically insignificant (Noorwali et al. 2018).

### 2.6 Inflammation and Oxidative Stress

Normal sleep, which is a psychophysiological process mediated by the central nervous system (CNS), plays a critical role in modulating the immune system through its effect on the distribution of immune cells and the production of inflammatory cytokines (Irwin 2023). Sleep deprivation disrupts the effector systems that regulate the immune system, leading to abnormal increases in inflammatory responses (Besedovsky et al. 2019). IL‐6 levels follow a circadian rhythm with peaks occurring at 7:00 P.M. and 5:00 A.M. As a result, night sleep is likely essential for the nocturnal increases in IL‐6 levels and synthesis of tumor necrosis factor (TNF). Sleep disorder reduces IL‐6 secretion and delays the increase of IL‐6 levels over the night (Irwin 2023).

Sleep disturbance alters the pattern of IL‐6 secretion, shifting it from nighttime to daytime, resulting in excessive IL‐6 production during the day (Vgontzas et al. 2005). Individuals with insufficient sleep experience reduced slow‐wave sleep (SWS) and increased REM sleep duration. Furthermore, sleep deficiency also decreases toll‐like receptor 4 (TLR4) stimulation and IL‐6 synthesis at night while increasing IL‐6 levels during the day (Bonilla‐Jaime et al. 2021). Sleep deprivation activates inflammatory signaling pathways, including nuclear factor‐κB (NF‐κB), activator protein 1 (AP‐1), and signal transducer and activator of transcription (STAT) family proteins. This leads to elevated mRNAs encoding pro‐inflammatory cytokines such as IL‐6 and TNF (Irwin 2019). Oxidative stress is characterized by an imbalance between ROS production and the antioxidant capacity of the cell (Demirci‐Cekic et al. 2022). ROS plays a significant role in the physiological effects of sleep deprivation. The existing evidence shows that sleep reduces oxidative stress (Noguti et al. 2013). Conversely, excessive production of pro‐inflammatory cytokines, oxidative stress, and neuronal damage disrupts circadian rhythm and reduces the quality and quantity of sleep. Sleep also promotes anti‐oxidative mechanisms (Scoditti et al. 2022). However, an animal study showed that total sleep deprivation increased antioxidant responses in several regions of the rat brain. This finding suggests that the observed response may be an adaptive mechanism to counteract oxidative stress caused by sleep deprivation (Ramanathan and Siegel 2011).

Additionally, SWA and REM (rapid eye movement) sleep can be increased through the intraventricular infusion of the oxidized glutathione (GSSG) (Honda et al. 1994). Short‐term sleep deprivation increases the antioxidant response in the brain and other organs, while prolonged sleep deprivation diminishes these responses, indicating that prolonged wakefulness causes chronic oxidative stress, which leads to the failure of antioxidant mechanisms to maintain the accumulation of pro‐oxidants (Atrooz and Salim 2020). Sleep deprivation also enhances neuronal firing, upregulates beta‐site APP‐cleaving enzyme 1 (BACE1) proteins, and exacerbates neuroinflammation and oxidative stress (Atrooz and Salim 2020; Mouton‐Liger et al. 2012). Adequate sleep quality and adequate sleep duration are associated with improved circulating biomarkers of oxidative stress (γ‐glutamyl transferase [GGT]) and antioxidant capacities (bilirubin, carotenoids, uric acid, vitamins A, C, D, and E), which play a crucial role in mediating the sleep duration (Kanagasabai 2016). Cherries containing polyphenols, melatonin, carotenoids, and vitamins E and C contribute to the antioxidant properties. The tart cherry extract also impacts oxidative stress and antioxidant levels by lowering the hydrogen peroxide (H_2_O_2_) levels caused by reactive oxygen species (ROS) and enhancing levels of glutathione peroxidase (Kelley et al. 2018; Mansoori et al. 2021). Tart cherries have been indicated to have several potential health benefits, including protecting against oxidative stress. Sheau C. Chai et al. examined the effect of tart cherry juice on biomarkers of inflammation and oxidative stress and demonstrated that consuming 480 mL of tart cherry juice for 12 weeks lowered malondialdehyde (MDA) and Ox‐LDL levels (Chai et al. 2019).

### 2.7 Tart Cherry and Inflammation

Long‐term dietary factors could change the inflammatory condition, which is closely associated with insomnia (Wirth et al. 2020). Several studies confirmed that sleep disorder is linked to inflammatory cytokines (particularly CRP and IL‐6) and glucocorticoids (Cholerzyńska et al. 2024; Zhao et al. 2020).

Bioactive compounds such as polyphenols with anti‐inflammatory properties are associated with the sleep–wake cycle. One of these ingredients is anthocyanin, which is found in red‐orange to blue‐violet food pigments (Do et al. 2023).

Tart cherry is rich in antioxidants, including aglycone cyanidin, one of the anthocyanin derivatives. Aglycone cyanidin exhibits anti‐inflammatory effects comparable to nonsteroidal anti‐inflammatory drugs (NSAIDs) such as naproxen (Coles 2012; Jariyapamornkoon et al. 2013).

Tart cherry mechanism included restricting the production of advanced glycation end products (AGEs), inhibiting NF‐κB, decreasing ROS production, increasing the expression and function of an enzymatic antioxidant system such as glutathione peroxidase and catalase (CAT), reducing cyclooxygenase (COX) activities, eliminating nitric oxide radicals, and decreasing monocyte chemoattractant protein 1 (MCP‐1). Furthermore, tart cherries' tryptophan and melatonin content can boost sleep outcomes and have a potential antioxidant capacity (Jariyapamornkoon et al. 2013; Karlsen et al. 2007; Sabou et al. 2021; Scoditti et al. 2012).

A placebo‐controlled study examined the effect of consuming cherry juice for 2 weeks on inflammatory conditions (240 mL 2 times/day). This study revealed that procyanidin B‐2 in cherry juice inhibited serum indoleamine 2,3‐dioxygenase (IDO), increased tryptophan availability, and reduced inflammation. Furthermore, this study found that procyanidin B‐2 at the dosage of 24–50 μM could inhibit IFN‐γ signaling pathways (Simper et al. 2019).

Tart cherry significantly reduced inflammation by lowering CRP and TNF‐α levels (Chai et al. 2019). These various effects of cherry on anti‐inflammatory function in different articles can be dose dependent.

In a randomized‐controlled trial involving 37 adults aged 65–80 years, participants consumed 480 mL of tart cherry juice daily for 12 weeks. This research showed that tart cherry juice significantly reduced levels of CRP, MDA, and OxLDL. However, no significant changes in TNF‐α, plasma 4‐hydroxynonenal (4HNE), plasma 8‐hydroxydeoxyguanosine (8‐OHdG), and nitric oxide (NO) were observed (Chai et al. 2019).

Similar effects have been observed with other food compounds rich in phytochemicals and antioxidants. A study showed that a daily oral dose of 500 mg resveratrol significantly reduced levels of IL‐12P40 and IL‐12P70 while increasing macrophage‐derived chemokine (MDC), interleukin (IL)‐4, fibroblast growth factor (FGF)‐2, and caused a change in ADL (ADCS‐ADL) scores after 52 weeks (Moussa et al. 2017). Karin Jacob et al. explored the effect of consuming 330 mL/day of tomato juice for 20 days on inflammatory marker levels in 106 overweight females and reported a significant reduction in IL‐8 levels (Ghavipour et al. 2013).

### 2.8 Neurotransmitters

Neurotransmitters (NTs) are biologically active chemicals that mediate electrochemical communication between neurons (Gasmi et al. 2022). The hypothalamus is an essential part of the brain that plays a significant role in sleep regulation. Specific groups of hypothalamic neurons and also adjacent groups of basal forebrain neurons produce the NT γ‐aminobutyric acid (GABA) (Moussa et al. 2017). Opposite to other NTs, the level of GABA during NREM sleep is higher than during wakefulness or REM sleep. GABA neurons can inhibit the activity of cells that are involved in wakefulness (McCarley et al. 1995). Various neurons, such as histamine, norepinephrine, serotonin, hypocretin, and glutamate, are directly inhibited by GABAergic sleep‐active neurons. This inhibition is linked to wakefulness (Siegel 2004).

The sleep–wake cycle is a complex physiological process that is regulated by changes in various NTs and neuromodulators including glutamate, acetylcholine, gamma‐aminobutyric acid (GABA), norepinephrine, dopamine, serotonin, histamine, hypocretin, melanin‐concentrating hormone, adenosine, and melatonin. It is worth noting that multiple interconnected NT systems within the central nervous system play a crucial role in the regulation of the sleep–wake cycle (Oh et al. 2019; Siegel 2004).

NT levels are influenced by dietary habits (McCarley et al. 1995). Serotonin, often referred to as “the happy molecule” plays a central role in mood regulation, appetite, social behavior, sexual drive, sleep, reminiscence, learning and gastrointestinal. The main sources of serotonin are fruits, vegetables, and seeds such as bananas, cherries, strawberries and tomatoes (Gasmi et al. 2022). The existing evidence shows that cherry is rich in phenolics, particularly anthocyanins, with potential anti‐neurodegenerative properties (Motohashi and Sakagami 2009). These Cherry phenolics protect neurons against cell‐damaging oxidative stress in a dose‐dependent manner which is mainly attributed to anthocyanins (Kim Dae‐Ok et al. 2005).

The most significant link between NTs and sleep is mediated by melatonin (Falup‐Pecurariu et al. 2021). Serotonin is first converted into N‐acetylserotonin, which is then methylated; it acts as a methyl donor for melatonin synthesis (Ahmad et al. 2023).

### 2.9 Melatonin

Melatonin, an indole compound also known as N‐acetyl‐5‐methoxytryptamine, was traditionally perceived to be secreted by the pineal gland. However, recent research showed that melatonin can be synthesized by non‐endocrine organs, including the skin, serotonin‐producing cells in the gastrointestinal tract, cerebellum, and immune system (Zisapel 2018).

The suprachiasmatic nucleus (SCN) regulates melatonin synthesis and secretion. In pineal cells, tryptophan undergoes hydroxylation and decarboxylation to make serotonin. N‐acetyltransferase shifts serotonin into N‐acetylserotonin, which is methylated by hydroxylindole‐O‐methyltransferase to produce melatonin (Masters et al. 2014; Poza et al. 2022).

Melatonin plays a key role in sleep regulation, serving as a signal of darkness that promotes sleep. Melatonin concentration increases in darkness and falls when exposed to light to provoke wakefulness (Vasey et al. 2021). While melatonin influences multiple cells in the body, its sleep‐promoting effects are mostly due to its response to the suprachiasmatic nucleus (SCN; the master clock), particularly on the melatonin receptors (Foster 2021; Masters et al. 2014). Melatonin binds to two main receptors, including MT1 and MT2, which are G‐protein‐coupled receptors and affect sleep and circadian rhythm. As a result, rich melatonin foods directly improve sleep. It is hypothesized that MT1 is associated with the hypnotic effects of melatonin, whereas MT2 is linked to the circadian rhythm regulation (Cardinali et al. 2022; Netzer et al. 2024).

The signaling pathway is a vital cellular mechanism involving multiple signaling cascades, such as Janus kinase, which plays a key role in the secretion of various signaling molecules and growth factors (Li et al. 2019). Melatonin significantly influences the JAK/STAT (Janus kinase/signal transducers and activators of transcription) signaling pathway by modulating immune cell activity, particularly macrophage, and thereby reducing inflammatory factors and activation of JAK/STAT pathways (Mihanfar et al. 2022).

Exogenous melatonin sources can enhance sleep quality in people with insomnia by helping them fall asleep faster, sleep longer, and feel more alert in the morning (Moon et al. 2022). Melatonin is found in sweet and tart cherries, with concentrations ranging from 10–20 ng/g in sweet cherries and 2.1–13.5 ng/g in tart cherries (Kanagasabai 2016). Tart Montmorency cherries are a rich source of plant chemicals, including melatonin, making them beneficial for regulating the sleep–wake cycle (Howatson et al. 2012).

One randomized, double‐blind, controlled crossover study investigated the effects of sour cherry juice on melatonin levels and sleep quality. Individuals consumed either a placebo or 60 mL sour cherry juice concentrate with a dose of *42.6 lg/30 mL per serving (*85.2 lg day^−1^). This study reported that consumption of sour cherry juice concentrate increased external melatonin levels and improved sleep duration (Howatson et al. 2012).

It is important to note that melatonin has a relatively short half‐life and its levels fluctuate significantly under the influence of external sources such as melatonin‐rich foods.

Chung et al. found that tart cherry juice intake could not change melatonin levels in female hockey players (Chung et al. 2022).

Similar effects have been observed with melatonin‐containing foods. In a trial, the administration of two different doses of saffron, 1 h before sleep, significantly increased blood melatonin concentration in the evening compared to a placebo (Lopresti et al. 2021).

In another study, consumption of 0.5–1 g of the *Pistacia vera* extract would be sufficient to receive 2–5 mg of melatonin. In addition, the extract appeared to enhance the effects of melatonin by activating Gi protein‐dependent pathways on melatonin receptors, improving symptoms of insomnia and sleep disorders (Labani et al. 2023).

## 3 Result

In this systematic review, six clinical studies were carried out to evaluate the effect of tart cherry‐containing compounds on sleep quality, melatonin secretion, and related indices of insomnia in different populations.

Five of the six studies reported significant improvements in the quality of sleep after drinking tart cherry or a supplement (Chung et al. 2022; Howatson et al. 2012; Langan‐Evans et al. 2023; Losso et al. 2018; Pigeon et al. 2010).

### 3.1 Sleep Quality

Howatson et al. (2012) reported that tart cherry juice consumption for 7 days increased sleep time, sleep efficiency, and time in bed (*p* < 0.05). Although the randomized, double‐blind, and crossover study design, along with objective (actigraphy) and biochemical (urinary melatonin) measurements, strengthens the validity of the results, the study lacks an examination of inflammatory or oxidative markers and has limited generalizability due to the small sample size and short intervention period. In Losso et al. (2018), sleep duration was increased by 84 min (*p* = 0.0182), and sleep productivity index improved (*p* = 0.03). The study design was robust, and the use of polysomnography as an objective measure increased the validity and accuracy of the study, but the very small sample size and short duration of the intervention period limited the statistical power of the study. Langan‐Evans et al. (2023) found that consumption of a mixture containing sour cherry powder resulted in a decrease in the onset of sleep (24 min; *p* = 0.002), an increase in sleep time (*p* = 0.01) and sleep efficiency (*p* = 0.03) but the small sample size (16 people) and short‐term intervention period (3 days) pose limitations in generalization and assessment of long‐term effects. Simper et al. (Simper et al. 2019) showed significant improvement in sleep initiation delay (*p* = 0.001), and other sleep parameters did not improve. Nevertheless, the lack of detectable melatonin in the studied supplement and the insignificant difference in tryptophan consumption between groups, along with the lack of a widespread effect on sleep parameters, have created limitations in explaining the mechanism of action and generalizing the results. Pigeon et al. (Pigeon et al. 2010), a significant decrease was observed in the wake of the onset of sleep (WASO) compared with placebo, while other indices such as sleep initiation time and sleep efficiency did not significantly differ from placebo; on the other hand, the small sample size and relatively moderate to weak effects, as well as the lack of significant improvement in objective sleep parameters (e.g., overall sleep and sleep efficiency) compared to placebo, have created limitations in proving the strong efficacy of this drink.

### 3.2 Melatonin Level

Melatonin secretion and biomarkers can relate to dormancy, and four studies were conducted to study changes in the level of melatonin and its metabolites; Howatson et al. (2012) showed a significant increase in the level of urinary 6‐sulfatoxymelatonin. Simper et al. (Simper et al. 2019) also reported an increase in the level of 6‐SMT in the urine after the supplement consumption containing tart cherry (*p* < 0.001); however, due to the lack of direct identification of melatonin in the compound, the role of tryptophan was emphasized. Langan‐Evans et al. (2023) also reported an increase in the metabolites associated with melatonin and physiological hormones such as D‐serine and L‐glutamic acid with a metabolomics approach. Chung et al. (Chung et al. 2022) did not show any significant difference in the level of melatonin and cortisol between sour cherry and placebo groups (interaction effect); however, the short‐term intervention period and limited sample size, lack of significant changes in melatonin and cortisol levels, and lack of complete control of factors affecting sleep have created limitations for explaining the mechanism and generalizing the results.

### 3.3 Inflammation and Oxidative Stress

Losso et al. (2018) found that tart cherry juice consumption significantly reduced the levels of prostaglandin E2 (PGE‐2), one of the most important inflammatory mediators. Also, the activity of the enzyme IDO, which is activated by stimulating the kynurenine pathway in response to inflammation, was reduced, and the kynurenine to tryptophan ratio was significantly reduced, indicating inhibition of the IDO‐dependent inflammatory pathway.

Nevertheless, several studies suggested several mechanisms for the effect of tart cherry on dormancy, including: increased availability of tryptophan and decreased kynurenine to tryptophan ratio (Losso et al. 2018) and inhibition of IDO enzyme activity by procyanidin present in tart cherry (in vitro), possible effect of foreign melatonin via tart cherry (Howatson et al. 2012).

## 4 Discussion

Table 1 presents the included articles. This review aimed to characterize and analyze the association between tart cherry supplements and sleep disorders. Sleep disorders are prevalent, disrupt the normal daily cycle, and negatively affect mental and physical health (Binks et al. 2020; Table 2).

**TABLE 1:** The included studies in the systematic review.

Author	Study design	Participants	Study duration	Sample size for control groups	Intervention	Effect size	Variables	Outcomes
Chung et al. (2022)	Randomized clinical trial	19 Field hockey players	5 Days	Placebo group (*n* = 9)	30 mL tart cherry juice	NR	1. Sleep quality recorded by actigraphy 2. Intermittent exhaustion exercise 3. Levels of melatonin and cortisol in the blood	In intervention groups: 1. Was not observed in the levels of melatonin (before: 21.81 ± 14.96, after 48 h: 21.45 ± 16.11) and cortisol (before: 15.21 ± 2.80, after 48 h: 12.50 ± 3.19). 2. Significant interaction effects total time in bed (TTB; *p* = 0.015), wake after sleep onset (WASO; *p* = 0.044), and movement index (MI; *p* = 0.031) variables.
Simper et al. (2019)	Double‐blind randomized clinical trial crossover	20 Participants (female *n* = 9)	7 Days	NR	Cherry extract with 200 mL of water every evening, approximately 1 h before going to sleep	Efficiency: 0.50 (−0.16, 1.17) Total sleep: 0.47 (−0.33, 1.30)	1. Urinary levels of 6‐sulphatoxymelatonin 2. Sleep quality 3. Physical activity levels	In intervention groups: 1. Increased 6‐SMT levels following nighttime recharge treatment (*p* < 0.001) 2. No effect on any sleep parameters with the exception of sleep latency (*p* = 0.001). 3. Significant difference in dietary tryptophan consumption between the intervention and placebo (*p* = 0.047)
JN Losso et al. (2018)	Double‐blind randomized clinical trial crossover	11 Healthy female or male	2 Weeks/separated by a 2‐week washout	NR	240 mL 2 times/day of tart cherry juice	NR	1. Sleep was evaluated by 5 validated questionnaires 2. Serum indoleamine 2, 3‐dioxygenase (IDO) 3. Kynurenine‐to‐tryptophan ratio 4. Prostaglandin E2 (PGE‐2)	In intervention groups: 1. Increased sleep efficiency on the Pittsburgh Sleep Quality Index (*p* = 0.03) 2. Inhibited IDO (*p* < 0.05) 3. Increased tryptophan availability (*p* < 0.05) 4. Increased sleep time by 84 min on polysomnography (*p* = 0.0182) 5. Decreased serum kynurenine‐to‐tryptophan ratio (*p* < 0.05)
Howatson et al. (2012))	Double‐blind randomized clinical trial crossover	20 Volunteers (men (*n* = 10) and women (*n* = 10))	7 Days	NR	Tart cherry juice concentrate (approximately 90–100 tart cherries and was diluted with approximately 200 mL of water)	NR	1. Sleep quality recorded by actigraphy 2. Urinary 6‐sulfatoxymelatonin	In intervention groups: 1. Significantly elevated total melatonin content (*p* < 0.05). 2. Significant increases in time in bed, total sleep time, and sleep efficiency total (*p* < 0.05).
Pigeon et al. (2010)	Double‐blind randomized clinical trial crossover	15 Older adults	8 Weeks	NR	Drink two 8‐oz servings of whole Montmorency tart cherries and apple juice processed to shelf‐stable conditions and provided by the manufacturer (CherryPharm)	Insomnia severity index: 0.55 (0.19–0.86) Sleep onset latency: 0.17 (0.07–0.23) Wake after sleep onset: 0.46 (−0.01–0.49) Total sleep time: 0.18 (0.47–0.59) Sleep efficiency: 0.18 (0.30–0.49)	1. Sleep continuity (sleep onset, wake after sleep onset, total sleep time, and sleep efficiency) was assessed by 2‐week mean values from daily sleep diaries 2. Disease severity by the Insomnia Severity Index	1. Statistically significant pre‐ to post‐treatment improvements on all sleep variables (Insomnia Severity Index (*p* < 0.05), sleep onset latency (*p* < 0.05), wake after sleep onset (*p* < 0.01), total sleep time (*p* < 0.01), and sleep efficiency (*p* < 0.05)). 2. Significant reductions in insomnia severity (minutes awake after sleep onset)
Langan‐Evans et al. (2023)	Double‐blind randomized clinical trial crossover	9 Males and 7 females	3 Days	NR	Intervention treatment composed of 1000 mg of tryptophan, 3000 mg of glycine, 300 mg of magnesium, 220 mg of tart cherry powder, and 200 mg of L‐theanine, with the placebo treatment containing 4720 mg of cellulose	NR	1. Assessment of urine samples 2. Measures Pittsburgh Quality Sleep Index, Core Consensus Sleep Diary, and Karolinska Sleepiness Scale survey tools, alongside objective actigraphy	1. Reduced sleep onset latency (−24 ± 25 min; *p* = 0.002) 2. Increased total sleep time (22 ± 32 min; *p* = 0.01) 3. Increased sleep efficiency (2.4% ± 3.9%; *p* = 0.03) 4. Reducing morning sleepiness (*p* = 0.02)

Abbreviations: 6‐SMT: 6‐sulfatoxymelatonin; DHA: docosahexaenoic acid; EPA: eicosapentaenoic acid; HRV: heart rate variability; IDO: indoleamine 2,3‐dioxygenase; NR: none reported; PGE2: prostaglandin E2; PSQI: Pittsburgh Sleep Quality Index; QOL: quality of life; TAC: total antioxidant capacity.

**TABLE 2:** Quality of included studies in the meta‐analysis.

Study, year (ref.)	Random sequence generation	Allocation concealment	Blinding of participants and personnel	Blinding of outcome assessment	Incomplete outcome data	Selective outcome reporting	Other sources of bias	Overall quality
Chung et al. (2022)	L	U	L	U	L	L	L	Fair
Simper et al. (2019)	L	H	L	U	L	L	L	Fair
Losso JN, et al. (2018)	L	U	L	L	U	L	L	Fair
Howatson et al. (2012)	L	U	U	L	H	L	L	Fair
Pigeon et al. (2010)	L	U	L	L	L	L	L	Good
Langan‐Evans et al. (2023)	L	U	L	U	L	L	L	Fair

Tart cherries are claimed to have several health benefits due to their high content of phytonutrients (Moon et al. 2022) and NTs, including melatonin and tryptophan, which regulate the sleep–wake cycle (Lopresti et al. 2021), reduce oxidative stress (Labani et al. 2023), and circulate inflammatory markers (Pigeon et al. 2010). Tart cherry influences the gut microbiome composition and contributes to controlling inflammatory markers and glucose modulation (Howatson et al. 2012). A study also showed that tart cherry juice consumption reduced insomnia severity index scores (Howatson et al. 2012).

The adverse cycle of sleep is associated with elevated TNF plasma levels that are commonly found in sleep apnea, insomnia, and sleep disorders (Traustadóttir et al. 2009). Pro‐inflammatory markers, including IL‐1β and TNF, enhance SWS (Kelley et al. 2006) while sleep disturbances are risk factors for inflammation (Hillman and Chrismas 2021). Tart cherry, rich in melatonin, plays a critical role in regulating the sleep–wake cycle in humans (Feuth 2024) and has a significant impact on the sleep cycle (Shoham et al. 1987). According to the study conducted by Chai SC et al. (Chai et al. 2019), the beneficial effect of tart cherries may be due to their antioxidant and anti‐inflammatory properties.

To the best of our knowledge, no systematic review study has established the relationship between inflammation, NTs, and sleep. This study filled this gap by investigating the potential (putative) anti‐inflammatory effects of long‐term tart cherry consumption on sleep disorders (Cholerzyńska et al. 2024). The results of current studies showed mixed results, which could be attributed to various doses and confounding factors considered in the analysis.

A study was conducted for 7 days in 2012 (Howatson et al. 2012), including 20 participants who reported that tart cherry extract diluted in water increased exogenous melatonin, which is beneficial in improving sleep duration and quality and managing disrupted sleep in healthy men and women. Despite the limitations of this study, it utilized cosinor analysis to track the circadian rhythm, providing insights into the short half‐life of melatonin and its fluctuations throughout the day.

In another study in 2018, a high dose of tart cherry juice (240 mL twice daily) increased tryptophan availability and reduced inflammation, which can improve insomnia (Losso et al. 2018). This suggests that tart cherry juice and its active ingredients may play a role in improving insomnia. Compliance was confirmed through elevated urinary 6‐SMT levels, reflecting the high tryptophan content of the supplement. Tart cherries may contribute to a small improvement in sleep disturbances in young healthy adults without diagnosed sleep problems. This result may be related to an increase in the endogenous production of melatonin via increased tryptophan intake.

Another study examined the impact of tart cherry juice on urinary levels of 6‐sulphatoxymelatonin (a marker of melatonin synthesis), sleep quality, and physical activity. In this trial, the improvement in sleep latency observed appears to be due to the supplement's tryptophan content (Simper et al. 2019).

A pilot study (Losso et al. 2018) examined the effect of a tart cherry juice beverage on the sleep of 15 older adults with insomnia over 2 weeks. The improvement in all sleep variables was found. The limitations of this study include the small sample size and the lack of polysomnographic assessment of sleep. Also, this study included elderly adults, who cannot be generalized to the general population.

A study (Pigeon et al. 2010) investigated nutritional modulation of sleep latency in 16 participants over 3 days. The intervention group received a combination of 1000 mg of tryptophan, 3000 mg of glycine, 300 mg of magnesium, 220 mg of tart cherry powder, and 200 mg of L‐theanine, while the placebo received 4720 mg of cellulose. This study reported increased sleep efficiency, leading to reduced perceptions of morning sleepiness. It should be noted that the individual or combined effects of the intervention cannot necessarily be extended to tart cherries.

One existing study (Chung et al. 2022) assessed 19 field hockey players over 5 days and demonstrated that tart cherry juice intake improved sleep quality, while no change in melatonin and cortisol levels was found. Consequently, it is proposed that tart cherry juice can help female athletes' quick recovery for future matches. The limitations of this study were that no participants experienced menstruation during the experiment. Also, various influential external factors were not considered.

However, dietary interventions such as melatonin, kiwi, tart cherry juice, or tryptophan‐rich diets can be effective in improving sleep quality naturally and without drug side effects (Doherty et al. 2019). Nevertheless, some studies show the opposite result (Noorwali et al. 2018). Human studies with more precise designs are needed to determine dosage, timing, and long‐term effects.

## 5 Strengths and Limitations

While this study employed a systematic approach, using a high‐sensitivity search strategy, several limitations should be considered for the current study. Firstly, there were variations in adjusted factors between the included studies. While an effort was made to make the literature search as exhaustive as possible, pertinent studies might have been missed. Secondly, there was a lack of access to some potentially eligible studies for full‐text screening. Thirdly, a meta‐analysis of findings was not performed. As a result, the conclusion remains limited to a qualitative synthesis. Lastly, repeat screening, selection of studies, data extraction, and quality assessment were not possible. Another limitation includes that review studies have been conducted with different doses, various types of intervention, and potential bias (e.g., publication bias) in various follow‐ups. It is suggested that future studies should include larger sample sizes, more standardized doses, and more diverse populations. On the other hand, to provide practical insights for clinicians, it would be better to conduct cellular studies to more precisely investigate mechanisms and dose–response studies.

In conclusion, this study provides direct evidence that dietary supplementation with tart cherry juice increases circulating NTs such as melatonin and tryptophan, which can decrease inflammation, enhance antioxidant capacity, and mitigate oxidative stress. These effects collectively improve sleep duration and promote the cycle of sleep. Variations in study outcomes may be attributed to differences in tart cherry juice dosages, which can influence their overall impact on sleep and general health.

## Author Contributions

**Fateme Barforoush:** writing – original draft (equal). **Sara Ebrahimi:** investigation (equal). **Shabnam Khademi:** investigation (equal). **Maryam Karimian Abdar:** investigation (equal). **Nava Morshedzadeh:** investigation (lead), methodology (equal), supervision (equal), writing – review and editing (equal).

## Ethics Statement

The authors have nothing to report.

## Consent

The authors have nothing to report.

## Conflicts of Interest

The authors declare no conflicts of interest.

## Data Availability

Data sharing is not applicable to this article as no datasets were generated or analyzed during the current study.
